# Development and application of a simple formate-buffered extraction method for the analysis of 104 organic pollutants in river suspended particulate matter by HPLC–ESI–MS/MS

**DOI:** 10.1007/s00216-025-06049-x

**Published:** 2025-08-07

**Authors:** Alexis P. Roodt, Ralf Schulz

**Affiliations:** 1 Eußerthal Ecosystem Research Station (EERES), RPTU Kaiserslautern-Landau, Birkenthalstrasse 13, D-76857 Eußerthal, Germany; 2 Institute for Environmental Sciences, RPTU Kaiserslautern-Landau, Fortstrasse 7, Landau, 76829 Germany

**Keywords:** Freshly deposited sediment, Suspended particulate matter, Organic micropollutant monitoring, Method development and validation, QuEChERS, Relative matrix effects

## Abstract

Globally, rivers are contaminated by a diverse range of organic micropollutants originating from waste water effluents or surface runoff in urban and agricultural areas. In rivers, suspended particulate matter (SPM), which can be constituted by a high proportion of organic material and fine mineral particles, sorbs many of these organic micropollutants and thus influences their distribution and residence times. Furthermore, benthic organisms, which live and feed in freshly deposited sediments, may be at increased risk of exposure to SPM-associated organic micropollutants. Efficient methods for the analysis of organic micropollutants in river SPM are therefore required for studying the distribution of these pollutants in space and time as well as the potential exposure of benthic organisms. In recent years, the “quick, easy, cheap, effective, rugged and safe” (QuEChERS) sample preparation methodology has gained increasing popularity for the analysis of organic micropollutants in environmental samples, including soils and sediments. In this study, we compared four extraction methods based on the QuEChERS methodology for their performance when combined with high-performance liquid chromatography tandem mass spectrometry analysis. As a result, a simple formate-buffered extraction method for the analysis of 104 selected organic pollutants, including pesticides and pharmaceuticals, in river SPM was developed and validated. The validated method was sensitive, with a median method limit of quantification of 1.0 ng/g. The method was applied to investigate the spatial and temporal distribution of the selected pollutants in SPM samples collected in the French-German border river, Lauter, which is an important regional refuge for biodiversity.

**Graphical Abstract:**

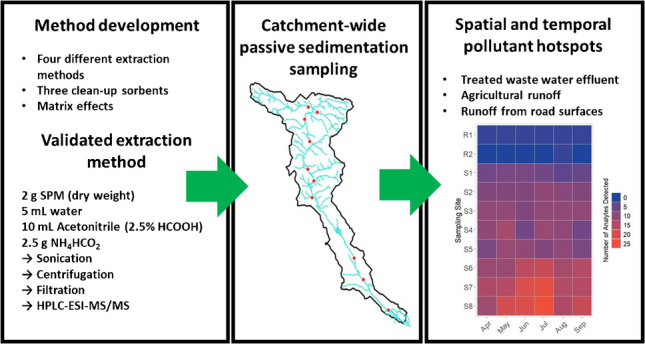

**Supplementary Information:**

The online version contains supplementary material available at 10.1007/s00216-025-06049-x.

## Introduction

Globally, diverse mixtures of synthetic organic micropollutants enter lotic ecosystems as a result of anthropogenic activities and contribute to the degradation of these aquatic habitats [1–3]. This includes pollutants entering via surface runoff from agricultural or urban areas such as pesticides [4] and tire-rubber-related pollutants [5], as well as pollutants present in waste water effluents such as components of personal care products and pharmaceuticals [3]. After entering the aquatic environment, organic micropollutants become distributed between environmental compartments including the dissolved aqueous phase and sorbed onto natural sorbents such as suspended particulate matter (SPM) [6]. Concentrations of organic micropollutants found in SPM are often higher than in bed sediments [7–9]. Furthermore, organic pollutants sorbed to SPM can be more resistant to natural biodegradation processes as well as transported and re-released following changes in hydrological conditions [10]. SPM and freshly deposited surface sediments can hence act as a contaminant sink with potential to negatively affect benthic organisms living and feeding in or on them [11]. SPM and their corresponding freshly deposited surface sediments therefore play an important role in the bioavailability, transport, distribution and residence times of organic micropollutants in lotic ecosystems [12]. Moreover, contaminated SPM can become a source of soil pollution with potential ramifications for terrestrial food webs when it is deposited on land during flood events [13].

Monitoring of organic pollutants in SPM and freshly deposited surface sediments is therefore acknowledged as an important aspect of water quality management and for the protection of lotic ecosystems, especially for hydrophobic organic pollutants characterised by moderate to high octanol/water partition coefficients (logK_ow_ ≥ 3) [14]. However, a recent review of 163 studies which included 3890 measurements of 904 organic pollutants covering a wide range of logK_ow_ values (− 4.6 to 19.1) in sediments found no relationship between the pollutants’ logK_ow_ values and reported environmental concentrations [15]. The relatively high concentrations of hydrophilic organic pollutants, primarily pesticides and pharmaceuticals with logK_ow_ < 3, found globally in sediments and SPM can be attributed to the large volumes of these chemicals produced, their incomplete removal by waste water treatment plants and their chronic discharge into the environment. Fluvial SPM is also a complex matrix which can contain a high proportion of organic material (e.g. mixtures of macromolecules containing both hydrophobic and hydrophilic domains) in addition to fine mineral particles (e.g. clays and silts) which can support the sorption of hydrophilic organic micropollutants [16–20]. The assessment of hydrophilic organic micropollutants such as pesticides and pharmaceuticals sorbed to SPM is therefore of growing interest and relevant for a comprehensive monitoring of organic pollutant distribution and exposure of benthic organisms in lotic ecosystems. The large number of hydrophilic organic pollutants which are potentially released into lotic ecosystems and the complexity of the SPM matrix, however, also poses a significant analytical challenge. This results in the need for the development of multi-target analytical methods which are able to reliably extract and measure concentrations of a wide range of analytes in SPM samples while also being easy and fast to perform, thus facilitating rapid sample throughput.

Currently, many published studies which have investigated organic micropollutants in sediments and SPM have extracted analytes from samples using organic solvents such as methanol or acetone in combination with accelerated solvent extraction and ultrasound-assisted extraction techniques [15]. However, the QuEChERS (quick, easy, cheap, effective, rugged and safe) sample preparation approach which was originally developed for the extraction of pesticide residues from fruits and vegetables has become increasingly popular in recent years for the extraction of a wide range of organic micropollutants in environmental samples, including soil and sediment [21, 22]. Newly developed extraction methodologies for organic micropollutants from primarily soil but also sediment using QuEChERS methodologies have shown good performance and are faster and simpler to implement in comparison to traditional sample preparation techniques such as accelerated solvent extraction [23]. There are three main versions of the QuEChERS method which are widely applied, namely the original unbuffered [24], the citrate-buffered [25] and the acetate-buffered [26] versions. Apart from the introduction of a buffer in the latter two versions, all three methods consist of the same three main steps. This involves (i) an initial salting-out and liquid–liquid extraction for which acetonitrile, magnesium sulfate (MgSO_4_), sodium chloride (NaCl) and buffer salts (when applicable) are added to a wet sample. After phase separation, a portion of the acetonitrile phase is (ii) transferred to a clean-up step involving dispersive solid phase extraction (dSPE). The cleaned extract is then (iii) analysed by liquid or gas chromatography coupled with mass spectrometry [22]. Further development of the QuEChERS methodology saw the replacement of the low vapour pressure MgSO_4_ and NaCl salts with a more volatile ammonium formate (NH_4_HCO_2_) buffer which was proposed as an alternative QuEChERS method to reduce salt deposits in mass spectrometers [27]. This version of the method performed comparably to the official AOAC 2007.01 acetate-buffered method when tested on food matrices but has not seen widespread usage in other applications such as the extraction of organic micropollutants from sediments [21].

An important aspect of developing new analytical methods is an appropriate validation which evaluates aspects of both sample preparation and instrumental analyses in order to define measurement limits and support the validity of the data generated using the method. Both soil and sediment are uniquely challenging matrices when developing and validating quantitative analytical methodologies due to their structural complexity, the potential compositional variability between samples along with various mechanisms of interaction between the matrix and analytes [18]. The complexity of these matrices thus requires special considerations during method validation. For example, insufficient interaction between analytes and the matrix prior to extraction of fortified samples can result in an overestimation of the performance of an extraction method [28]. Similarly, structural changes to the matrix caused by lyophilization prior to solvent extraction can potentially restrict access of organic solvents to analytes [18]. This can result in an underestimation of the fraction of extractable analytes in the original samples if not considered during the validation [23]. Furthermore, the complexity of soil and sediment matrices provides multiple mechanisms of sorption of organic compounds (e.g. hydrophobic, electrostatic or aromatic pi-system interactions) by endogenous components of the matrix, the impact of which can depend on both the extraction conditions (e.g. pH, solvent polarity or ionic strength) as well as the organic compound’s concentration [18, 29]. The potential for concentration-dependent sorption of analytes by the matrix should therefore also be considered when determining extraction recoveries and method sensitivity limits. Furthermore, coextracted endogenous components of the sample matrix can influence the accuracy of analyte measurements through matrix effects. For example, when matrix components alter the efficiency with which analytes become ionised during analysis by high-performance liquid chromatography coupled to mass spectrometry by electrospray ionisation (HPLC–ESI–MS/MS) [30]. The impact of compositional variability of the sample matrix between samples and the resulting variability in matrix effects should therefore also be considered during the method validation [31].

The overall objective of this study was to develop and validate a rapid and simple method for the extraction and analysis of a wide range of analytes, including pesticides, pharmaceuticals and personal care products, from samples of passively collected SPM by HPLC–ESI–MS/MS. The validated method was then used to investigate the spatial and temporal distribution of the targeted analytes in SPM samples collected over a six-month period from a tributary of the river Rhine in southwestern Germany. The investigated river (Lauter/Wieslauter) is of special interest because it forms the border between France and Germany and it is impacted by both urban and agricultural pollutant sources, but also flows through protected natural areas and provides an important regional refuge for biodiversity, for example, for juvenile endangered Atlantic salmon [32].

## Materials and methods

### Chemicals, reagents and equipment

Analytical standards (purity ≥ 98%) were purchased as multicomponent solutions (100 mg/L) from Restek (Bellefont, PA, USA) or as single-component standards from Sigma-Aldrich (St. Louis, MO, USA) and Dr. Ehrenstorfer (Augsburg, Germany). A full list of reagents and equipment used in this work is provided in the supplementary information (Table S1).

### Sampling sites, sample collection and pre-treatment

All sampling was conducted between April and October 2023 as part of the Interreg project RiverDiv, which focused on water resource management and biodiversity protection under climate change in the transnational Lauter river between Germany and France. The Lauter river can be divided into the upper and lower courses. The upper course (called the Wieslauter) is located within the Palatinate Forest–North Vosges Biosphere Reserve. The catchment of the upper course of the river is therefore primarily dominated by forested and small urban areas (Fig. S1). The lower course of the river flows through the town of Wissembourg, after which it forms the French-German international border ending at the river Rhine. The catchment of the lower course includes a greater proportion of agricultural and urban land usage compared to the upper course but is also bordered for approximately 40% of its course on the German side of the river by a recognised nature reserve, namely the Lauterniederung. Samples were collected at eight sampling sites (S1–S8, Table S2) which were located downstream of discharges from nine waste water treatment plants of various sizes (population equivalents of 150–30,000, Table S3) which are located within the river catchment. Sampling site S1 was located closest to the river source and site S8 closest to the river mouth. A further two sites were selected as reference sites (R1 and R2, Table S2, Fig. S1) and were located in headwater tributaries of the Lauter, which had no upstream agricultural or waste water inputs.

Samples of freshly deposited surface sediment derived from SPM (from here on referred to as SPM samples) were collected monthly between May and October 2023 (6 samples per site) by using passive sedimentation traps which were constructed in-house, based on a design published elsewhere [33]. Briefly, a PVC-plastic tube with diameter of 8.5 cm and length of 35.5 cm was fitted with polypropylene-plastic funnels at both open ends. The funnel openings had a diameter of 1.2 cm, allowing water to flow through the trap at a reduced flow velocity relative to the ambient flow velocity which facilitated sedimentation of SPM inside the trap. The passive sedimentation traps were bound to metal rods embedded in the riverbed and were oriented parallel to the water flow at approximately 60% of the water depth. The depth of the sedimentation traps in the water column was adjusted occasionally during the sampling period due to seasonal variation in the river depth. The passive sedimentation traps were emptied at 28- to 36-day intervals (Table S4). Upon collection, the entire passive sedimentation trap was removed from the river and a SPM sample was collected by straining the contents of the trap through a filter paper with a pore size of 20 µm to separate the sample from excess water. The collected SPM samples were then transferred to polyethylene plastic bags which were placed on ice and transported to the laboratory. In the laboratory, the SPM samples were then transferred to 50-mL polypropylene tubes and centrifuged at 170 RCF for 5 min. The supernatant was subsequently decanted and the SPM sample pellets were then frozen at − 20 °C before being freeze-dried. Dried SPM samples were sieved (mesh size 1 mm) and stored at − 20 °C prior to extraction and analysis. It must be noted that the passive sampling technique provided flow-velocity weighted samples in which light and fine grain sized particles may not be completely collected.

### Organic matter content and pH of SPM samples

The percentage organic matter content (%OM) of the SPM samples was measured by using a loss-on-ignition method when sufficient dry weight (dw) was available. Samples were prepared by weighing 5.0 ± 0.5 g subsamples of the freeze-dried and sieved SPM sample material into ceramic dishes. The samples were then combusted at 550 °C for 5 h before being re-weighed. The %OM was then calculated from the weight lost using the following equation:

$$\%\;\mathrm{Organic}\;\mathrm{matter}=\frac{{\mathrm{Weight}}_{\mathrm{dry},\;\mathrm{pre}\;\operatorname{combustion}}-{\mathrm{Weight}}_{\mathrm{post}\;\operatorname{combustion}}}{{\mathrm{Weight}}_{\mathrm{dry},\;\mathrm{pre}\;\operatorname{combustion}}}\times100$$

The %OM determined for individual SPM samples is provided in the supplementary information (Table S4). The overall average ± standard deviation of the measured values for %OM across all collected samples was 18.3 ± 8.0 (*n* = 53).

The pH values of freeze-dried SPM samples were measured for samples when sufficient sample weight was available. The pH was measured with a pH meter after rehydrating in a 0.01 M calcium chloride (CaCl_2_) solution with a 1:10 w/v ratio. The pH values determined for individual samples are provided in the supplementary information (Table S4). The overall average ± standard deviation of pH values across all samples was 5.8 ± 0.4 (*n* = 55).

### Reference SPM material

For method development, a large amount of reference SPM material was prepared by pooling SPM sample material from two reference sites located in headwater tributaries of the river Lauter, which were not impacted by waste water effluents or agricultural land use (Table S2). This SPM reference material had an OM content of 18% and a pH of 6.2, which was within the range of values determined for SPM samples across all sampling sites (Table S4).

### Selected analytes and HPLC–ESI–MS/MS analyses of SPM sample extracts

All SPM sample extracts (extractions described in the following sections) were analysed by high-performance liquid chromatography-electrospray ionisation-tandem mass spectrometry (HPLC–ESI–MS/MS). The HPLC separation was performed using a reverse phase column and the MS/MS analysis was performed using a triple quadrupole mass spectrometer. The separation and mass spectrometer acquisition parameters had been optimised on the instrument and used in previous studies [34, 35]. The relevant instrument parameters are provided in the supplementary information (Table S5). The targeted multiple reaction monitoring (MRM) acquisition method included a total of 104 analytes (Table S6) which were selected based on either their usage and ubiquity in the environment or their emerging relevance.

The selected analytes included 36 fungicides, 28 herbicides and 24 insecticides/biocides as well as three relevant metabolites of these. Of these pesticides, 67 were approved for use in the European Union at the time of sample collection [36]. The registration of the remaining 21 pesticides had expired up to 7 years before the sampling, but were included because of their continued detection in European surface waters [35]. The selection of analytes also included 10 pharmaceuticals and one relevant metabolite thereof, which are either frequently detected in surface waters [3] or of emerging relevance [37]. For example, two protease inhibitors were included, which were prescribed for the treatment of COVID-19, but for which environmental monitoring data is still scarce. Additionally, the tire-rubber-related pollutant 6PPD-quinone (6PPD-Q) was also included due to its emerging relevance and a scarcity of environmental monitoring data in Europe [38, 39]. Chromatographic and mass spectra generated by the analyses were evaluated using MassHunter Workstation quantitative analysis software for QQQ (version 10.0). A single analyte, namely 6PPD-Q, was present in the reference SPM material. To compensate for this background signal, the “quantitate with blank subtraction” add-in in the MassHunter software was used during data analysis.

### Initial method parameters and preliminary SPM-extraction experiments

A series of preliminary extraction experiments were performed by comparing analyte signals in extracts of fortified samples, which were prepared using the reference SPM material. For these experiments, 2.0-g portions of freeze-dried SPM material were fortified by pipetting 50 µL of a mixture of all 104 analytes in acetonitrile onto the surfaces of the SPM material, which provided a final concentration of 5.0 ng/g based on dry weight. The solvent was then allowed to evaporate for 30 min before beginning the extractions.

Preliminary analyte recoveries were compared between four extraction methods. The simplest method involved the extraction of dry SPM material with 10 mL of acidified acetonitrile (AA) containing 2.5% (v/v) formic acid. The remaining three methods were modified versions of existing QuEChERS methods that included an initial rehydration of the dried sample material and a buffered extraction. Two of these methods were based on the official QuEChERS methods, namely the acetate-buffered AOAC 2007.01 method (Q-AB) and the citrate-buffered CEN Standard EN 15662 method (Q-CB) which used MgSO_4_ and NaCl in addition to the respective buffer salts during the extraction. The fourth extraction method was based on a formate-buffered (FB) extraction that does not include any salts in addition to the buffer. In all three buffered extraction methods, the dried samples were initially rehydrated by vortexing them for 30 s in 5 mL of water before 10 mL of acetonitrile was added. In the case of the Q-AB and FB methods, the added acetonitrile contained 1% (v/v) acetic acid and 5% (v/v) formic acid, respectively. After the addition of the acetonitrile in all four methods, the samples were vortexed for 10 s and, in the case of the three buffered methods, the extraction salts were added. For the Q-AB method, 4.0 g of MgSO_4_ and 1.0 g of sodium acetate were added. The Q-CB method received 4.0 g MgSO_4_, 1.0 g NaCl, 0.50 g sodium citrate dibasic sesquihydrate and 1.0 g sodium citrate tribasic dihydrate. The FB method received 2.5 g of NH_4_HCO_2_. After the addition of the extraction salts, the samples were vortexed once more for 10 s. Samples from all four methods were then placed in an ultrasonic bath for 20 min at 20–25 °C. Afterwards, the samples were centrifuged at 4150 RCF for 5 min at 20 °C. A 1-mL aliquot of the supernatant from each sample was then filtered using 0.2-µm PTFE syringe filters into brown glass HPLC vials prior to analysis by HPLC–ESI–MS/MS.

### Dispersive solid phase extraction (dSPE) clean-up

The effect of including a dSPE clean-up on analyte recoveries was tested using the extracts obtained using the four extraction methods (AA, Q-CB, Q-AB and FB). For this, the preliminary recovery experiment was performed as described in the previous section; however, 1-mL aliquots of the extract supernatants were transferred to 2-mL tubes containing dSPE sorbents. Three dSPE clean-up sorbents were evaluated for their effects on the analyte recoveries, namely, primary secondary amine (PSA), graphitised carbon black (GCB) and octadecyl silica (C18). PSA and C18 were tested at a concentration of 25 mg/mL, and GCB at a concentration of 6 mg/mL. The dSPE tubes containing sorbent and sample extracts were vortexed for 30 s before being centrifuged at 4150 RCF for 5 min. The supernatants were then filtered and analysed as described previously for the uncleaned extracts.

### Matrix effects

Matrix effects on the MRM-signal intensities of individual analytes were assessed by comparing the slopes of calibration series prepared in pure solvent and in SPM sample extracts with concentrations corresponding to sample concentrations of 0.0, 5.0, 12.5, 25.0 and 50.0 ng/g (dw). Matrix effects on individual analytes were evaluated using the following equation:

$$\mathrm{Matrix}\mathrm{effect}\;\left(\%\mathrm{ME}\right)=\left(\frac{{\mathrm{Slope}}_{\mathrm{Extract}}}{{\mathrm{Slope}}_{\mathrm{Solvent}\;}}\times100\right)-100$$

Positive and negative values of %ME thus indicated signal enhancement and suppression, respectively.

An assessment of %ME was initially performed for each of the four tested extraction methods (AA, Q-CB, Q-CB, FB) using the reference SPM material. The overall impact of matrix effects was compared between extracts by calculating the median absolute deviation (MAD) using the %ME for each analyte across all 104 analytes.

In addition to evaluating the %ME, the total amount of coextracted matrix material in the acetonitrile phases of the extracts was gravimetrically determined in extracts produced using the four tested extraction methods (AA, Q-CB, Q-CB, FB) by transferring the acetonitrile phase to a pre-weighed polystyrene weighing boat and reweighing after allowing the solution to evaporate at approximately 20 °C overnight.

### Comparison of accuracy and precision between extraction methods

The accuracy and precision of each extraction method were assessed by determining the average percentage recoveries (%RECs) and percentage relative standard deviations (%RSDs) of analytes extracted from replicate fortified samples prepared using reference SPM material (*n* = 4). For this purpose, fortified samples were prepared by pipetting 50 µL of an acetonitrile solution containing a mixture of all 104 targeted analytes onto the surface of the samples to obtain a final sample concentration of 25.0 ng/g (dw). In comparison to the initial recovery experiments, a greater interaction between the SPM matrix and the analytes was required. For this reason, 2 mL of MS-grade water was added to the dry sample material after addition of the 50 µL fortification solution, and the samples were then vortexed for 30 s. The samples were then centrifuged at 4150 RCF for 5 min before being left to stand at room temperature (approximately 20 °C) in complete darkness for 1 h. The sorption of organic pollutants by natural sorbents (such as soils) is often characterised by a rapid initial uptake phase followed by a slower uptake phase which can require between several hours and several days before equilibrium is reached [18]. In this experiment, however, the equilibration time was limited to 1 h in order to minimise the impact of analyte degradation or the formation of non-extractable residues, which would add a further level of complexity to the interpretation of the recovery results. After the 1-h standing period, samples were frozen at − 40 °C before being freeze-dried over 24 h. The fortified samples were then extracted using each of the four extraction methods as described in the previous section.

### Final extraction method, method validation and measurement quality criteria

Field-collected SPM samples were extracted using a formate-buffered method (FB) without applying a dSPE clean-up step. For this method, 5 mL of water was added to 2.0 g portions of freeze-dried SPM sample material in 50-mL polypropylene tubes. The samples were then vortexed for 30 s and 10 mL of acetonitrile containing 5% formic acid was added. The samples were subsequently vortexed for 10 s before 2.5 g of NH_4_HCO_2_ was added. After addition of the salt, the samples were vortexed once more for 10 s and then placed in an ultrasonic bath for 20 min at 20–25 °C. Samples were then centrifuged at 4150 RCF for 5 min. After centrifugation, a 1-mL aliquot from each sample was filtered using 0.2-µm PTFE syringe filters into a brown glass HPLC vial.

The performance of the final extraction method and HPLC–ESI–MS/MS analysis was validated in terms of specificity, linearity, accuracy, precision, instrument limits of quantification (ILOQs), method limits of quantification (MLOQs) and relative matrix effects. The specificity of the method was supported by acquiring two characteristic MRM transitions for each analyte (Table S6), by reviewing the ratios of qualifier-to-quantifier MRM-signal intensities, and reviewing the chromatographic retention times. For the positive identification of analytes in samples, the ratios of qualifier-to-quantifier MRM-signal intensities were required to be within 20% compared to the analytical standard. Deviations in chromatographic retention times were required to be within 0.05 min. The linear response range of the instrument was determined by preparing a matrix-matched calibration series in reference SPM material corresponding to sample concentrations of 0.10, 0.20, 0.30, 0.40, 0.50, 0.75, 1.0, 1.5, 2.0, 2.5, 5.0, 10, 50 and 250 ng/g (dw). As an assessment of calibration linearity, the back-calculated analyte concentrations in the measured calibration series were required to be within 20% of the actual concentrations based on a linear regression weighted by the reciprocal of the predictor (1/*x*). The lowest concentration in this series was defined as the ILOQ and was required to have signal-to-noise (S/N) ratios of at least 10 and 3 for the quantifier and qualifier MRMs, respectively. The S/N ratios were determined using a root mean square (RMS) algorithm with a noise standard deviation multiplier set to 5 in the MassHunter quantitative analysis software.

The accuracy and precision of the method were assessed by determining the %REC and %RSDs of replicate (*n* = 4) fortified samples. For this purpose, fortified samples were prepared using the method described in the previous section at seven fortification levels (0.25, 0.50, 1.0, 2.5, 5.0, 12.5 and 25 ng/g, dw). The %RECs and %RSDs of the measured analyte concentrations were required to be between 70 and 120% and below 15%, respectively. MLOQs were defined as the lowest concentration in fortified samples which fell within the linear range and satisfied the criteria for S/N, accuracy and precision. Analyte signals in field-collected samples that fell below the respective MLOQ but still satisfied the criteria for specificity and S/N ratios were reported as “ < MLOQ”. Relative matrix effects were evaluated using the calibration slope method to investigate the variability in %ME due to varying %OM in reference samples. For this purpose, calibration series were prepared in extracts from reference sites R1 and R2 (sampling dates: 6.7.23, 7.9.23 and 4.10.23). Additionally, the calibration slope methodology was used to evaluate the efficacy of using the reference SPM material for a matrix-matched calibration by using the gradient of calibration slope as the denominator in the %ME calculation (%ME in previous section). For the quantitative measurements of analytes, the limit to %ME was set at ± 20%. Results for analytes which did not meet the quality criteria for %ME were reported qualitatively. Additionally, a bracketing calibration was performed to monitor response drift over the course of sample analysis with a limit for the difference between the slopes of the bracketing calibration series set at 30%.

## Results and discussion

### Initial extraction method parameters

At the onset of the extraction method development, suitable extraction parameters as well as sample and extraction solvent amounts which were to be used for all further method development were determined. A 10–15 g sample weight and a sample-weight to solvent-volume ratio of one-to-one is used in the official versions of the QuEChERS methodology which were originally developed for high moisture content samples [24, 40]. Applications of the QuEChERS methodology to dry samples therefore require water to be added prior to solvent extraction. The high %OM of the dried SPM samples (average %OM: 18.3, Table S4) however required a large volume of water (> 10 mL) to create a semi liquid mixture and visible phase separation when using a 5–10 g sample during the liquid–liquid extraction. For this reason, the sample amount was reduced to 2 g which was rehydrated with 5 mL of water. The volume of acetonitrile used to extract the samples was kept at 10 mL because an excess of solvent has been reported to improve results for applications of QuEChERS methodologies to soil matrix and can reduce matrix effects by diluting coextracted endogenous substances [23, 41]. Ultrasonication of the extracts after solvent addition was also included from the onset of method development because this has been shown to improve the extraction of organic pollutants from soil when applying QuEChERS extraction methods [23].

### Preliminary extraction recoveries and dSPE clean-up

The four tested extraction methods (AA, Q-CB, Q-AB and FB) all performed very similarly in terms of preliminary recoveries (fortification performed without rehydrating and refreezing the samples after analyte addition). Across all four methods, 89–96% of the analytes had preliminary recoveries within the defined acceptable range (70–120%) without the inclusion of a dSPE clean-up (Table S7). The inclusion of a dSPE clean-up using either PSA, GCB or C18 had overall negative effects on analyte recoveries and reduced the total number of analytes falling within the acceptable range by up to 15%. The inclusion of GCB resulted in reduced recoveries for the largest number of analytes and C18 the least. These results were consistent with the known negative effects of these sorbents on recoveries of specific analytes. For example, GCB reduced the recoveries of structurally planar pesticides by up to 40%, and PSA negatively impacted alkali-sensitive analytes [21, 40]. However, none of the tested dSPE sorbents provided a great improvement in analyte signal responses compared to the uncleaned extracts by removing matrix components responsible for signal suppression during the ESI process (Table S7). Limited utility of dSPE clean-up procedures has similarly been found by other researchers who have tested dSPE clean-up procedures as part of QuEChERS-based methodologies for multiresidue analyses of pesticides in soil using HPLC–ESI–MS/MS [31, 34, 42, 43]. A dSPE clean-up was therefore not included in further method development in the present study, which had the added advantage of reducing the overall complexity of the final method.

### Comparison of accuracy and precision between extraction methods

**Fig. 1:**
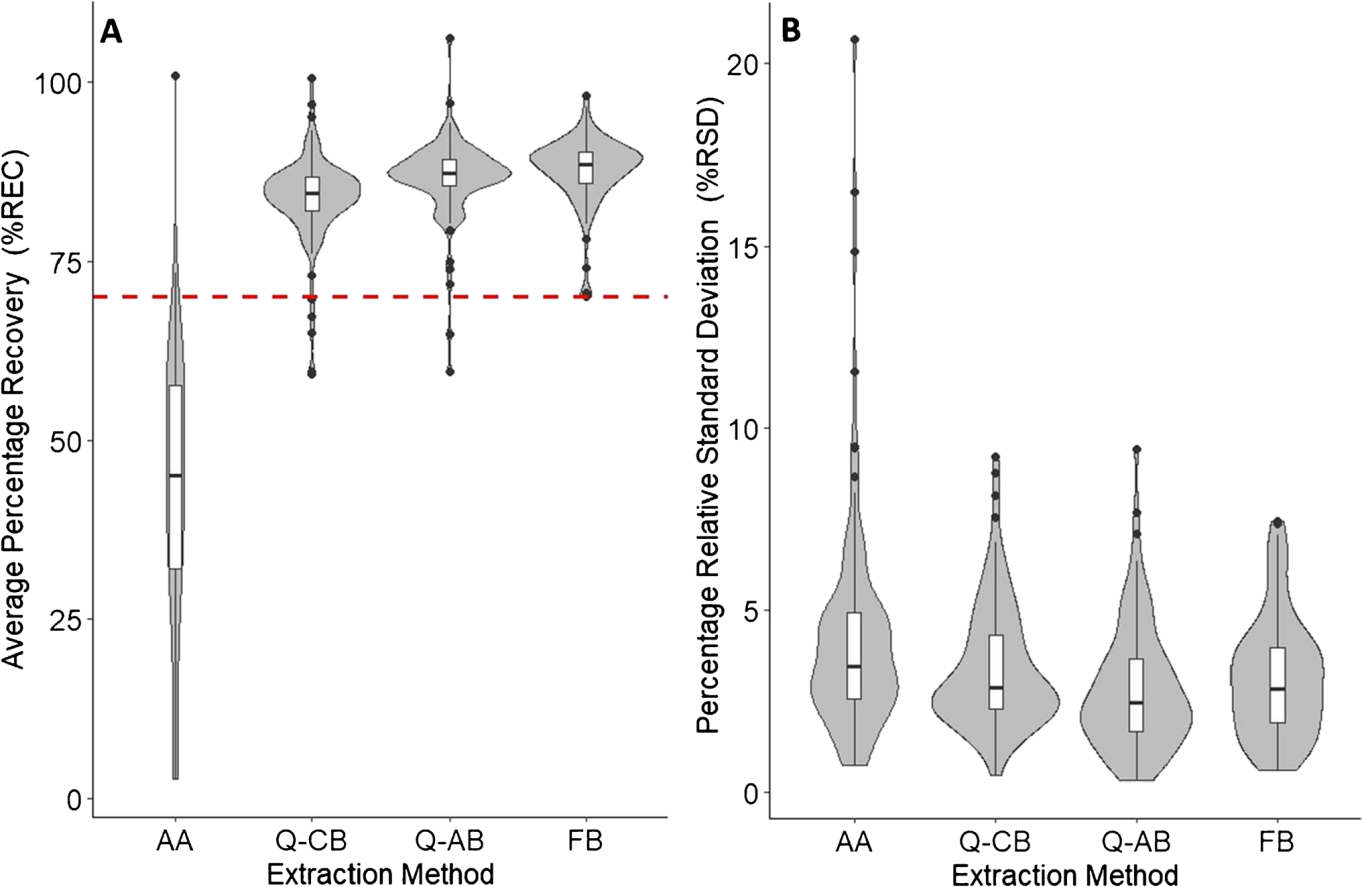
Average (*n* = 4) percentage recoveries (%RECs, **A**) and percentage relative standard deviations (%RSDs, **B**) of 104 analytes when extracted from fortified reference SPM sample material (25.0 ng/g, dw) after ageing (1 h) of fortified samples using an acidified acetonitrile extraction (AA), formate-buffered (FB) extraction or modified versions of the official citrate (Q-CB)- and acetate (Q-AB)-buffered official QuEChERS methods

Allowing time for the analytes in fortified samples to interact with the hydrated SPM matrix before subsequent re-drying and extraction of the fortified samples had a substantial impact on the determined %RECs. The AA method, which did not include an initial rehydration step during the extraction, performed the worst out of the four tested methods and provided considerably lower %RECs compared to the initial recovery experiment (Fig. 1A, Tables S7 and S8). After allowing the analytes time to interact with the sample matrix, the %RECs for approximately 95% of analytes were below the threshold value of 70% compared to only 5% during the preliminary determination of %RECs (Table S8). The AA extraction method also performed slightly worse in terms of precision compared to the remaining three extraction methods, potentially due to greater variability in the penetration of the dry SPM matrix by the extraction solvent (Fig. 1B, Table S8). The three remaining buffered extraction methods, which included an initial rehydration step as part of the extraction, performed similarly in terms of both accuracy and precision. For these three methods, the median %RECs increased in the order Q-CB < Q-AB < FB, with a total of 95–100% of the analytes having %RECs within the range of 70–104% and RSDs < 10% (Fig. 1A, B, Table S8).

Better performance in terms of accuracy and precision for methods which included an initial rehydration step compared to the AA method is consistent with what has been found by other researchers who have developed extraction methods using acetonitrile for the quantitative determination of various classes of pesticides from dried soil material [21, 23]. The benefits of rehydrating soil samples during an extraction are considered to be due to the ability of water to better penetrate the dry soil structure compared to organic solvents [23]. Additionally, water molecules may have a greater ability to compete with polar analytes for adsorption sites on the matrix surface in comparison to less polar organic solvents [18]. In the current work, a trend towards increasing analyte %REC with increasing analyte lipophilicity (logK_ow_ values: − 2.9 to 6.9) was only observed when using the AA extraction method (Fig. S2). This observation appears to support the role of water in disrupting interactions between polar analytes and the SPM matrix, while hydrophobic interactions between less polar analytes and the SPM matrix can be disrupted to a greater extent by acetonitrile alone. These results corroborate both the importance of allowing analyte-matrix interactions to be established in the fortified SPM samples before evaluating extraction performance, as well as the necessity of a rehydration step in the extraction procedure, similar to what has been described in soil.

The observed differences in %RECs between the three buffered extraction methods in the present results (Fig. 1A) can be influenced by both the pH and ionic strength of the aqueous phase during the liquid–liquid partitioning. The experimental determination and comparison of aqueous phase pH between the three extraction methods is complicated by the presence of insoluble MgSO_4_ and residual acetonitrile in the aqueous phase; however, all three buffered methods are buffered in a similar range (pH 4–5) but differ in the ionic strength of the aqueous phase [40]. The slightly higher %RECs obtained when using the Q-AB method compared to the Q-CB method is similar to what has been found by other researchers who have compared the two official versions of these QuEChERS extraction procedures for the extraction of various pesticides from soil and sediment [43, 44]. The similar performance found between the Q-AB and FB methods in the present study also reflects what has been previously found for 43 pesticides (four of which were included in the present study) in food matrices when comparing a formate-buffered QuEChERS extraction to the official AOAC 2007.01 QuEChERS method [21]. Furthermore, the replacement of the exothermic dissolution of MgSO_4_ with the endothermic dissolution of NH_4_HCO_2_ may also have contributed to the slightly higher %RECs obtained when using the FB method compared to the Q-AB method due to decreased thermal decomposition of some target analytes.

### Matrix effects

Matrix effects during HPLC–ESI–MS/MS analyses are caused by coeluting matrix components that cause changes in analyte ionisation efficiencies during the ESI process and hence changes in analyte MRM-signal intensities relative to the MRM-signal intensities in the absence of matrix [45]. Both the concentrations of coextracted matrix material in SPM extracts and %ME on analyte MRM-signal intensities were therefore compared between the four extraction methods (AA, Q-CB, Q-AB and FB). Overall, matrix-induced signal suppression was the most commonly observed matrix effect and affected 70–80% of the 104 analytes across all four tested methods (Table S9). The median %ME was closest to null (− 1.6%) when using the AA method and between − 4.1 and − 4.3% for the remaining three methods (Fig. S3, Table S9). Furthermore, the median absolute deviations (MADs) of the %MEs were between 2.7 and 2.9% and therefore very similar between the AA, Q-CB and Q-AB methods, but largest (4.3%) when using the FB method (Table S9). The AA method was therefore the overall least impacted and the FB method the overall most impacted by matrix effects. These results also correlated with the gravimetrically determined concentrations of coextracted matrix material in each extract (0.53, 1.01, 0.67 and 2.08 mg/mL for the AA, Q-CB, Q-AB and FB methods, respectively).

Among the four tested methods, the AA method was the only method which did not include the addition of water and a liquid–liquid phase separation during the extraction. The smaller magnitudes of %ME when using the AA method may therefore be attributed to the poor solubility of some matrix components (e.g. proteins or salts) in pure acetonitrile compared to acetonitrile which contains some water [46, 47]. Moreover, the partitioning of polar matrix components into the acetonitrile phase in a liquid–liquid extraction is influenced by both the ionic strength of the aqueous phase and the amount of water in the acetonitrile phase [24, 40]. The Q-AB and Q-CB methods—which performed similarly in terms of both the magnitudes of %ME and the total amounts of coextracted matrix material—both included a combination of NaCl and excess MgSO_4_ in the extraction procedure to reduce the aqueous phase volume and induce phase separation between the water and acetonitrile phases. The FB method, on the other hand, relied solely on the ionic strength of NH_4_HCO_2_ in solution to induce phase separation, without excess magnesium sulfate. The lack of MgSO_4_ in the FB method may thus have resulted in the greater solubility and partitioning of some matrix components into the acetonitrile phase and hence greater probability of matrix effects occurring when using this extraction method.

Overall, the magnitudes of the observed %ME using all four methods were similar to what has been found by other authors who have developed similar methods for the analysis of a wide range of pesticides in extracts from agricultural soils by HPLC–ESI–MS/MS [31, 34]. Furthermore, the observed %ME across all four methods could be considered minor for between 97 and 100% of the analytes in the present study based on a threshold of 20% for both signal suppression and enhancement (Fig. S3). A threshold of 20% is the benchmark used by the SANTE 11312/2021 guidance document on *Analytical quality control and method validation procedures for pesticide residue analysis in food and feed* [48]. According to this guidance document, exceedances of the 20% threshold should be addressed in the calibration, which can be achieved by use of an appropriate matrix-matched calibration (see “Final method validation”).

### Final method validation

Based on the results of our experiments, an extraction using the FB method without a dSPE clean-up was chosen to undergo the final validation before being used for the analyses of field-collected samples. Using this method, the ILOQs were determined to be between 0.10 and 5.0 ng/g with a median of 0.50 ng/g (Table S10) and MRM-signal responses were linear up to the highest calibration level used (250 ng/g). Furthermore, the extraction method provided MLOQs between 0.25 and 25 ng/g with a median of 1.0 ng/g and MLOQs ≤ 2.5 ng/g for 80% of the 104 analytes (Tables S10 and S11). The values for ILOQ and MLOQ are similar to quantification limits published by other authors who have developed methods for the analysis of a wide range of organic pollutants including pesticides and pharmaceuticals in sediments [49–53]. However, it must be noted that various approaches for defining and determining analyte quantification limits are used by different authors. The criteria which we used to define the MLOQ included all steps in sample extraction (for example, lyophilization of samples after sample fortification) in addition to analyte MRM-signal criteria. This approach is important for reducing the risk of over- or underestimation of the method performance due to the complexity of the SPM matrix and its interactions with analytes [54, 55]. For example, the consideration of analyte extraction recoveries as part of the MLOQ definition was especially relevant for five analytes in the present work namely, trimethoprim, sulfamethoxazole, propamocarb, O-desmethylvenlafaxine and Omethoate. The ILOQs for these analytes were between 0.10 and 1.0 ng/g; however, the extraction method did not fulfil the criteria for accuracy below a fortification concentration of 25 ng/g (Table S10). The comparatively high MLOQs for these analytes could therefore be the result of their incomplete partitioning between the aqueous and acetonitrile phases or the result of non-linear concentration-specific interactions between these analytes and components of the SPM matrix.

### Relative matrix effects and matrix-matched calibration

**Fig. 2:**
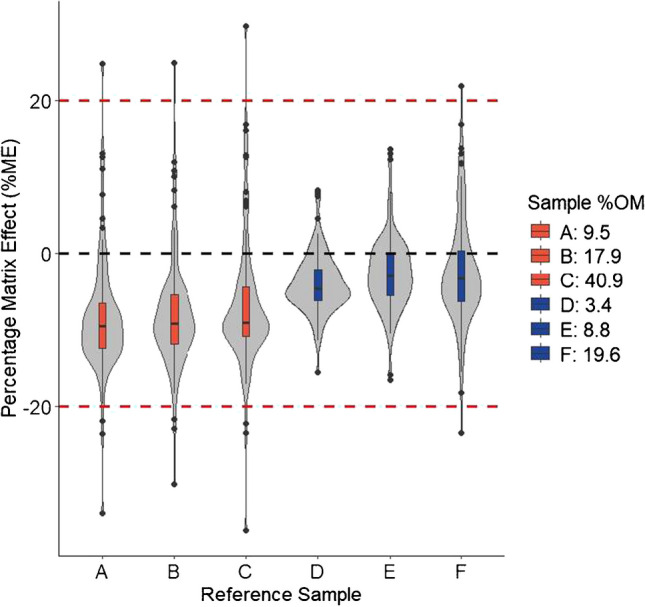
Percentage matrix effects (%ME) determined for 104 targeted analytes in extracts of six reference samples (**A**–**F**) which were prepared using a formate-buffered (FB) extraction method. The samples were collected at two reference sites (reference site R1 and R2 in red and blue, respectively) and contained increasing organic matter contents (%OM) between samples from the same site

Differences in matrix composition and thus %ME between different samples of the same type (relative matrix effects) can be a source of error in quantitative HPLC–ESI–MS/MS methods [56]. For example, %ME for a wide range of organic pesticides in extracts of agricultural soils is influenced by the organic carbon content of the soil, with samples which contain more organic carbon also having greater %ME [31]. Therefore, in addition to the assessment of %ME in extracts of the reference SPM sample material (see previous section, “Matrix effects”), we compared the %ME between extracts from six SPM samples which were collected from the two reference sites included in the present study (Table S2). The %OM of the individual samples was used as a proxy for matrix complexity, and the six SPM samples were therefore selected to be representative of the full range of %OM values (5.9–35.2) measured in SPM samples collected across all the sampling sites included in the study (Table S4). Evaluation of the %ME across all six reference SPM samples revealed that the variability in %ME (calculated MADs, Table S12) increased with increasing %OM in samples from both reference sites. Furthermore, between 70 and 90% of MRM-signal intensities of the 104 targeted analytes were affected by matrix-induced signal suppression across all six samples (Table S12). The magnitudes to which the analyte MRM-signals were suppressed were, however, consistently greater at reference site R1 compared to reference site R2 (overall median %ME-values of − 9.2 and − 3.6, respectively) despite overlapping ranges of %OM values between the samples from the two sites (Fig. 2, Table S12). Furthermore, the differences in the median %ME between the two sites were consistent for samples collected from the same site and correlated with consistent differences in the pH values (1.0–1.3 pH units) in the SPM samples collected from the two sites (Table S12). The consistent differences in SPM pH values between sites provide evidence for differing mineral or natural OM composition in samples between the sites. Our results therefore indicate that the composition of the OM in each sample, in addition to the amount, may play an important role in differences in %ME between samples.

Overall, the %ME were within ± 20% across all six reference samples for 100 of the 104 analytes (Fig. 2, Table S12). The MRM signals of the remaining four analytes, etofenprox, fenpyroximate, prosulfocarb and terbuthylazine were suppressed or enhanced by between 20 and 40% in at least three of the six reference samples (Fig. 2, Table S12). The efficacy of using the reference SPM material to prepare a matrix-matched calibration to compensate for the observed relative matrix effects between samples was therefore evaluated by recalculating the relative matrix effects using the slope of a matrix-matched calibration as the reference (Table S13). Overall, the use of the matrix-matched calibration reduced the impact of site-specific matrix-induced MRM-signal suppression and increased the overall median %ME values from − 9.2 to − 4.4 and − 3.6 to 0.5 for reference sites R1 and R2, respectively (Tables S12 and S13). Additionally, the use of a matrix-matched calibration reduced the %ME on prosulfocarb and terbuthylazine to within ± 20%. The matrix-matched calibration, however, did not compensate for matrix effects on the quantification of etofenprox and fenpyroximate and increased the %ME to greater than 20–40% for two additional analytes, namely carfentrazone-ethyl and flonicamid (Table S13). Based on these results, a matrix-matched calibration was used for the quantitative analysis of the 100 of the 104 targeted analytes in extracts of field-collected SPM samples. Results for the four analytes which did not meet the quality criteria for %ME when using the matrix-matched calibration were hence reported qualitatively.

### Application to field-collected SPM samples

**Fig. 3:**
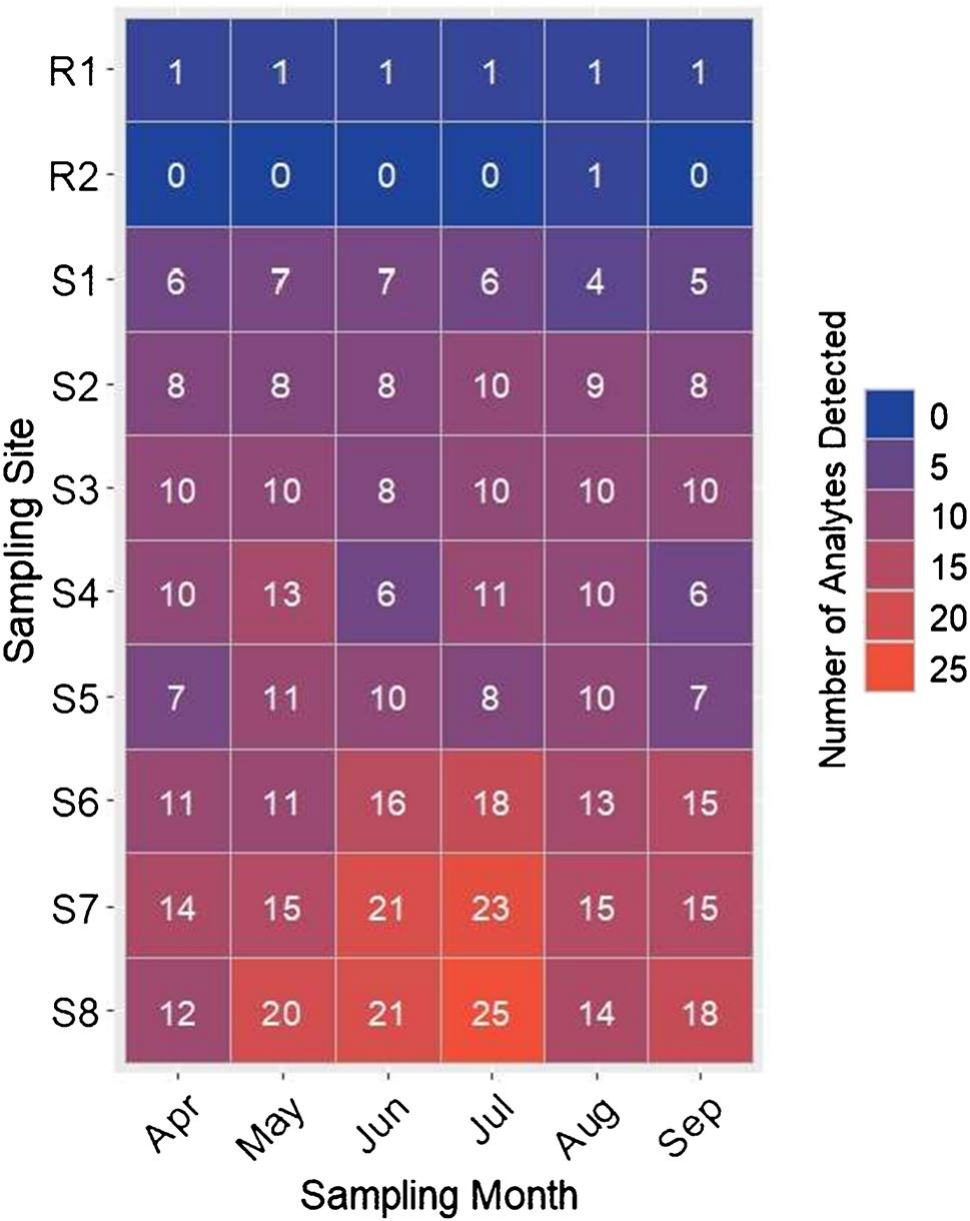
Total number of analytes detected (out of 104) in monthly (April–October 2023) SPM samples collected at eight study sites (S1–S8) and two reference headwater tributaries (R1 and R2) in the Lauter river catchment

The validated FB extraction method was used to investigate the spatial and temporal trends of the targeted organic micropollutants in the river Lauter, which is a tributary of the upper Rhine River and a regional refuge for biodiversity in southwestern Germany [57]. Of the 104 targeted analytes included in the analyses, 32 were detected in the monthly SPM samples collected over the 6-month sampling campaign (Table S14). Each of the SPM samples collected from the eight sampling sites contained between 4 and 25 analytes (Fig. 3), 19 of which were present at concentrations above their respective MLOQs (Table S14).

Overall, the pharmaceuticals, donepezil, carbamazepine, citalopram, trimethoprim, venlafaxine and its metabolite O-desmethylvenlafaxine were among the most frequently detected analytes and were found in over 70% of SPM samples across all eight sampling sites (Table S14). Three pharmaceuticals, namely, carbamazepine, citalopram and venlafaxine, were the most frequently quantified analytes and were detected above their respective MLOQs in 70–78% of the SPM samples. The highest concentrations across all sampling sites were consistently measured for the two antidepressants, citalopram (average: 9.0–47 ng/g) and venlafaxine (average: 1.0–12 ng/g), followed by the anticonvulsant carbamazepine (average: < MLOQ–4.0 ng/g) (Fig. S4). The ubiquity and relatively high concentrations of these pharmaceuticals in the SPM samples are consistent with global trends due to their widespread usage and incomplete removal during waste water treatment [15, 20, 58]. The measured concentrations of citalopram and venlafaxine were within the same range of concentrations measured in SPM samples collected from the Rhine River during the period 2006–2015 [20]. Moreover, the maximum measured concentrations for citalopram, venlafaxine and carbamazepine in the present study are within the 70th, 84th and 57th percentiles of globally published concentrations in sediments, respectively [15]. As a result of their pseudo-persistence in aquatic environments, these pharmaceuticals have also been widely detected in aquatic biota, which may be associated with sublethal behavioural effects on benthic organisms [58–60].

**Fig. 4:**
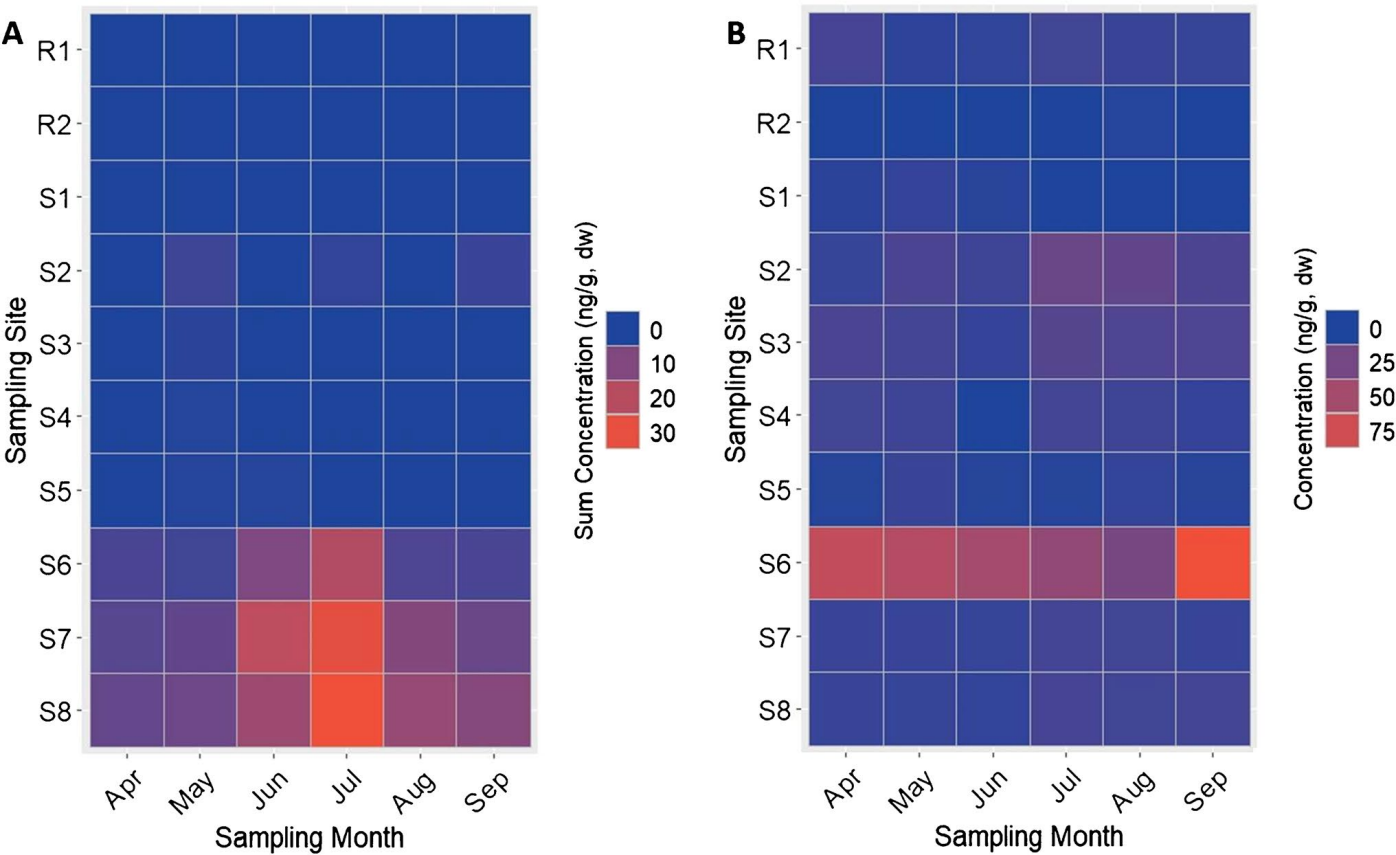
Sum concentrations of pesticides (**A**) and concentration of the tire-rubber-related pollutant 6PPD-quinone (**B**) measured above their respective MLOQs in SPM collected at 2 reference headwater tributaries and 8 study sites along the river Lauter

In addition to the pharmaceuticals, several pesticides were also frequently detected and quantified in the SPM samples. However, these analytes had more distinct temporal dynamics compared to the pharmaceuticals (Table S14). The total number of pesticides and the sum-pesticide concentrations peaked during the summer months (June–July) in the lower reach of the river (S6–S8) which is also closest to agricultural land use in the catchment (Fig. S1). SPM samples collected at these sites during this timeframe contained mixtures of pesticides consisting primarily of fungicides (3–11 analytes per sample) and herbicides (1–5 analytes per sample) with sum-pesticide concentrations between 9.0 and 32 ng/g, which consisted of over 80% fungicides (Table S14, Fig. 4A). All of the detected pesticides, with the exception of the legacy herbicide isoproturon, were registered for use in the European Union at the time of sampling. The peak concentrations of fungicides and herbicides in the SPM samples are consistent with the peak application period and peak water concentrations measured in small agricultural streams in Germany between May and June [61]. Furthermore, detection of complex mixtures of fungicides is consistent with their frequent prophylactic application and mitigation strategy for fungicide resistance development [62]. The presence of mixtures of especially fungicides in SPM and surface sediments has the potential to disrupt the rate of leaf litter decomposition by affecting the diversity of non-target aquatic fungi communities [63].

The tire-rubber-related pollutant 6PPD-Q was overall the most ubiquitous analyte in the SPM samples and was the only analyte detected in samples collected from the two headwater reference sites (Table S14, Fig. 3). Three-quarters of all SPM samples contained concentrations of 6PPD-Q between 1.0 and 10 ng/g. However, the highest concentrations of 6PPD-Q were consistently measured at site S6, which was the sampling site nearest downstream of the largest urban settlement in the river catchment (Fig. S1). The temporal dynamics of 6PPD-Q in samples over the 6-month sampling period at S6 also correlated with regional precipitation patterns (Table S15). A delayed peak concentration of 97 ng/g was measured in September after an increase in regional precipitation during August following a drier summer period between June and July (Fig. 4B). Our results are therefore consistent with studies which have attributed peak 6PPD-Q contamination of aquatic environments to road-surface runoff and weather conditions [64–67]. The role of precipitation makes concentrations of 6PPD-Q in surface waters very dynamic and challenging to monitor, with peak concentrations often occurring during storm events [65, 67–69]. Although most studies focus on aqueous concentrations, 6PPD-Q is a mid-polarity molecule with a predicted octanol–water partition coefficient (logK_ow_) of 3.98 and predominantly neutral charge at ambient pH values, which can thus facilitate its sorption to surface sediments and SPM [69, 70]. The collection and analysis of SPM samples may therefore provide an advantage over water grab samples when investigating the spatial and temporal dynamics of 6PPD-Q in aquatic environments over longer timeframes. Recently, 6PPD-Q has also been detected in sediment grab samples from two large and highly urbanised rivers in China, with maximum concentrations up to 46 ng/g [70, 71]. Our results therefore suggest that 6PPD-Q concentrations in SPM and surface sediments may also be substantial in smaller rivers bordered by smaller urban areas, especially following seasonal weather patterns and sudden increases in precipitation. Information on the risks of exposure to 6PPD-Q for benthic organisms is, however, currently limited. Since its first identification in 2020, knowledge on the toxicity of 6PPD-Q for aquatic organisms has rapidly advanced. Acute mortality due to dissolved 6PPD-Q can occur at environmentally relevant concentrations for very sensitive aquatic species, but extends over several orders of magnitude even among closely related species [72–74]. There is, however, emerging evidence that 6PPD-Q may bioaccumulate in aquatic organisms and may be associated with sublethal effects, which could have negative implications at the population level [75].

## Conclusion

We developed and validated a simple ultrasound-assisted formate-buffered liquid–liquid extraction method for the analysis of 104 organic pollutants in SPM samples by HPLC–ESI–MS/MS. The method, which relies solely on the ionic strength of an ammonium formate buffer in solution to induce phase separation between water and acetonitrile phases, has the advantage of being simple and fast, allowing for rapid throughput of samples. The method achieved good performance in terms of accuracy and precision at low analyte concentrations (median MLOQ: 1.0 ng/g, dw) when extracting analytes from SPM samples with relatively high %OM content. In addition to the %OM content of samples, the origin and therefore likely the composition of the samples were found to affect the degree to which matrix effects affected quantitative results, primarily by matrix-induced MRM-signal suppression. Overall, the validated method provided quantitative results for 100 of the 104 targeted organic micropollutants in SPM and could therefore be used to investigate the potential exposure of benthic organisms to these pollutants. The newly validated method is very simple to implement and has an advantage over older extraction methods which use more complex laboratory equipment and more chemicals. The rapid throughput of samples for analysis of pollutants from various sources also facilitates environmental monitoring studies in which spatial and temporal hotspots of pollution can be very dynamic, thus requiring large numbers of samples to be analysed. As a proof of concept, the method was used to generate relevant insights into the spatial and temporal distributions of 32 organic micropollutants in SPM samples collected from a river catchment of high conservation value in southwestern Germany.

## Supplementary Information

Below is the link to the electronic supplementary material.

ESM 1 (DOCX 1.92 MB)

ESM 2 (XLSX 105 KB)

## Data Availability

The authors confirm that the data supporting the findings of this study are available within the article and its supplementary materials.
