# Supplementary material for: Development and application of a simple formate-buffered extraction method for the analysis of 104 organic pollutants in river suspended particulate matter by HPLC–ESI–MS/MS
Source: Anal Bioanal Chem. 2025 Aug 7;417(23):5287–301. doi: 10.1007/s00216-025-06049-x (PMC12432068; doi:10.1007/s00216-025-06049-x)
Supplement: Supplementary file 1 — (DOCX 1.92 MB) [file 216_2025_6049_MOESM1_ESM.docx]

**Supplementary Material for:**

**Development and application of a simple formate-buffered extraction method for the analysis of 104 organic pollutants in river suspended particulate matter by HPLC-ESI-MS/MS**

**Alexis P. Roodt^1,2^*, Ralf Schulz^2,1^**

**^1^ Eußerthal Ecosystem Research Station (EERES), RPTU Kaiserslautern-Landau, Birkenthalstrasse 13, D-76857, Eußerthal, Germany**

**^2^ Institute for Environmental Sciences, RPTU Kaiserslautern-Landau, Fortstrasse 7, 76829 Landau, Germany**

***Corresponding author: roodt.a@rptu.de**

**Table of Contents**

**Fig. S1.** Map of the study area showing sampling sites in relation to nature protected areas and land cover in the Lauter catchment**S2**

**Fig. S2.** Average percentage recoveries of 104 targeted analytes measured by HPLC-ESI-MS/MS plotted against their respective log octanol/water partition coefficients (logP)**S3**

**Fig. S3.** Comparison of percentage matrix effects (%ME) determined for 104 analytes in extracts prepared from reference SPM-sample material using four extraction methods.**S4**

**Fig. S4.** Monthly (April – October 2023) concentrations of the three most frequently quantified pharmaceuticals, namely citalopram, venlafaxine and carbamazepine in SPM samples collected at the eight study sites along the river Lauter**S5**

**ReferencesS5**


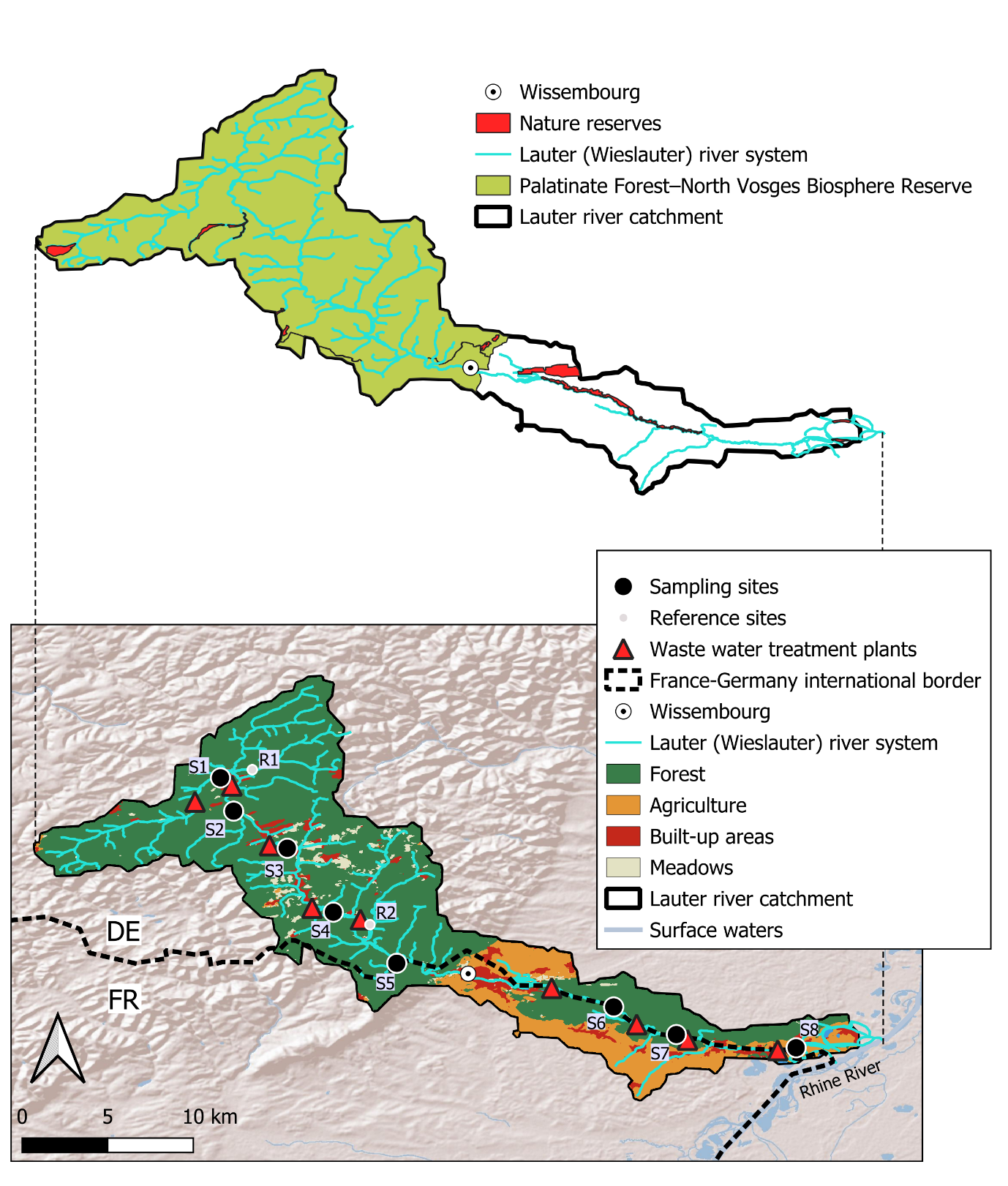


**Fig. S1.** Map of the study area showing sampling sites in relation to nature protected areas (above) and land cover (below) in the Lauter catchment (FR: France, DE: Germany). Land cover data for the year 2023 was obtained from the Sentinel-2 10m land use/land cover time series of the world [1].


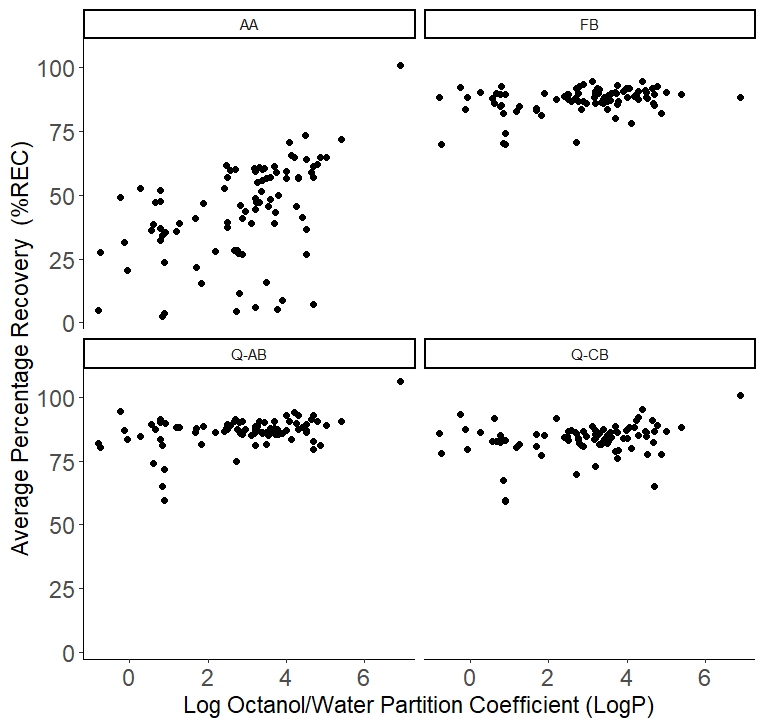


**Fig. S2.** Average (n = 4) percentage recoveries of 104 targeted analytes measured by HPLC-ESI-MS/MS plotted against their respective log octanol/water partition coefficients (logP). Analytes were extracted from reference SPM material fortified at a concentration of 25 ng/g (dry weight) using acidified acetonitrile extraction (AA), formate buffered (FB) extraction or modified versions of the official citrate (Q-CB) and acetate (Q-AB) buffered QuEChERS methods.


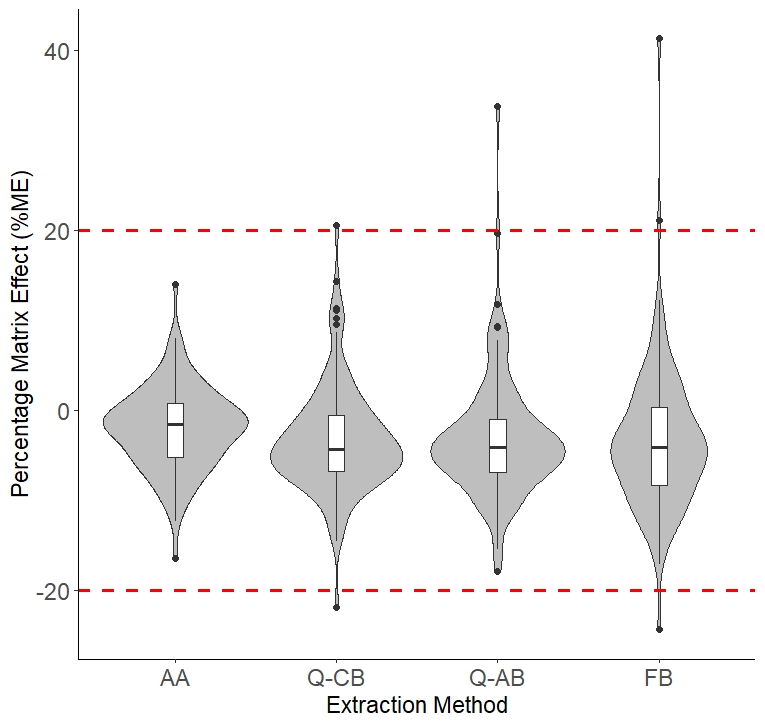


**Fig. S3.** Comparison of percentage matrix effects (%ME) determined for 104 analytes in extracts prepared from reference SPM-sample material using acidified acetonitrile extraction (AA), formate buffered (FB) extraction or modified versions of the official citrate (Q-CB) and acetate (Q-AB) buffered QuEChERS methods.


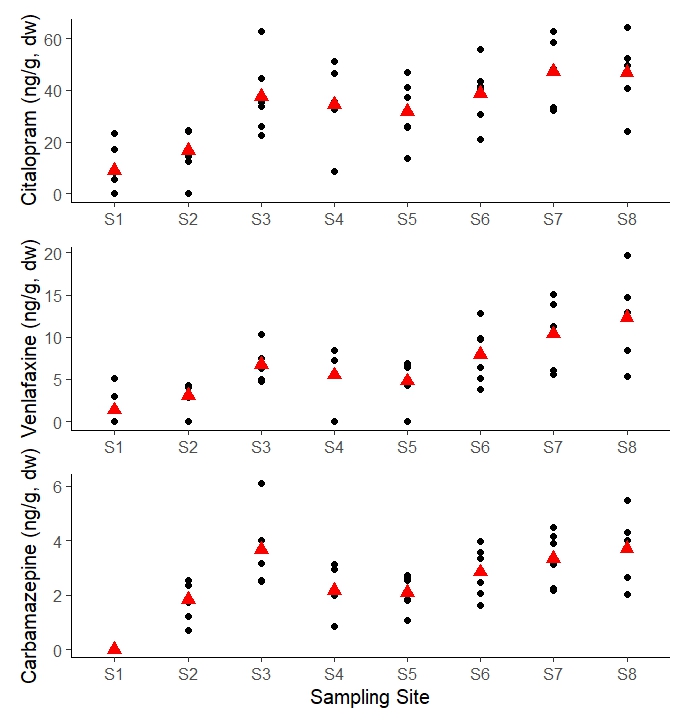


**Fig. S4.** Monthly (April – October 2023, n=6) concentrations (black points) of the three most frequently quantified pharmaceuticals, namely citalopram, venlafaxine and carbamazepine in SPM samples collected at the eight study sites (S1 – S8) along the river Lauter (Site numbering starts closest to the river source). Red triangles indicate the site-specific average analyte concentration.

**References**

[1] K. Karra, C. Kontgis, Z. Statman-Weil, J.C. Mazzariello, M. Mathis, S.P. Brumby, Global land use / land cover with Sentinel 2 and deep learning, in: 2021 IEEE Int. Geosci. Remote Sens. Symp. IGARSS, 2021: pp. 4704–4707.
